# Red Light-Dose or Wavelength-Dependent Photoresponse of Antioxidants in Herb Microgreens

**DOI:** 10.1371/journal.pone.0163405

**Published:** 2016-09-27

**Authors:** Giedė Samuolienė, Aušra Brazaitytė, Akvilė Viršilė, Julė Jankauskienė, Sandra Sakalauskienė, Pavelas Duchovskis

**Affiliations:** Laboratory of Plant Physiology, Institute of Horticulture, Lithuanian Research Centre for Agriculture and Forestry, Babtai, Lithuania; Islamic Azad University Mashhad Branch, ISLAMIC REPUBLIC OF IRAN

## Abstract

The purpose of this study was to evaluate the role of 638-nm and 665-nm LEDs on changes of antioxidants of basil (*Ocimum basilicum*) and parsley (*Petroselinum crispum)*, and to assess the effect of light quality on antioxidative status. Plants were grown in peat substrate for 19 days (21/17 ±2°C, 16 h). Experiments were performed in (I) a controlled-environment: B_455_,R_638_,R_665_,FR_731_(control); B_455_,R*_638_,R_665_,FR_731_; B_455_,R_638_,R*_665_,FR_731_; R_638_; R_665_ (B–blue, R- red, FR–far-red light). *PPFD* was set from 231 during growth, upto 300 μmol m^-2^ s^-1^ during 3-day treatment changing R_638_ or R_665_ *PPFD* level; in (II) greenhouse (November): high-pressure sodium lamps (HPS) (control—300 μmol m^−2^s^−1^); and HPS + 638 (HPS generated 90 and red LEDs—210 μmol m^−2^s^−1^). In general, under supplemental or increased red 638 nm light, amounts of tested antioxidants were greater in basil, whereas sole 665 nm or sole 638 nm is more favourable for parsley. Increased or supplemental red light significantly increased contents of phenolics, α-tocopherol, ascorbic acid and DPPH^•^ but suppressed accumulation of lutein and β-carotene in basil, whereas an increase of β-carotene and DPPH^•^ was observed in parsley. Hereby, the photoresponse of antioxidant compounds suggests that photoprotective mechanism is stimulated by both light-dose-dependent and wavelength-dependent reactions.

## Introduction

Basil and parsley differ in phytochemicals and are interesting because of the antioxidant capacities, such as β-carotene, lutein, phenolic compounds [1], α-tocopherol, and ascorbic acid [2]. The antioxidant activity of natural antioxidants is mainly based on their redox properties; these properties can play a key role in absorbing and neutralizing free radicals, quenching singlet and triplet oxygen, and decomposing peroxides [3]. However, internal (such as genotype) [4, 5] or external (such as environmental conditions) [6–8], factors may drastically influence the quantity or quality of the aforementioned phytochemicals. A few studies have shown that physiological responses may be caused by mild photooxidative stress [9, 10], as well as by light spectral quality [6, 8, 11]. It has been reported that accumulation of phytochemicals may become altered when phytohrome convert from an inactive (at 600 nm) to an active form (at 730 nm) [12]. In contrary to photosynthesis, which is a light-dose-dependent reaction, photoresponse is more a wavelength-dependent reaction [13]. Red and blue light are important for the optimization of photosynthesis, the action spectrum of biosynthetic wavelengths for the production of plant pigments differs between chlorophylls–which strongly absorbs in the red (663 and 642 nm) and blue (430 and 453 nm) region of the spectrum and carotenoid pigments, lutein and β-carotene—which absorb in blue region (448 and 454 nm) [14]. Previous researchers has demonstrated that phenolics content can become enhanced by supplemental red light [6, 9, 15]; antioxidant activity can become altered by red LED light [8, 9, 15, 16]; and far-red LED light can suppress antioxidant potential [8]. Carotenoids were enhanced with blue LEDs, but suppressed with far-red LEDs [6]. Additionally, blue LEDs improve ascorbic acid accumulation [17], however the response to red treatments is diverse [9, 16]. Thus, it kas been shown that metabolic changes as well as morphological and physiological responses of different plant are critically regulated by light. Technology based on solid-state light-emitting diodes (LEDs) allows for the selection of desired combinations of spectral components and required photosynthetic photon flux density (*PPFD*) [18]. In this way, certain light qualities and quantities may be chosen for specific plant, horticultural, pharmaceutical or culinary demands.

The purpose of this study was to evaluate the role of 638 and 665 nm red light components on quantitative changes of antioxidants and to assess the effect of light quality on the antioxidative status of basil and parsley.

## Materials and Methods

### Chemicals

2,2–diphenyl–1–picrylhydrazyl (DPPH) (Sigma-Aldrich, Germany), Folin-Ciocalteau reagent (Fluka, Germany), Na_2_CO_3_ (Sigma-Aldrich, Germany), ascorbic acid (Penta, Check rep.), oxalic acid (Fluka, Germany), methyl viologen (Sigma-Aldrich, Germany), alpha tocopherol (Supelco, PA, USA), lutein and β-carotene (Sigma-Aldrich, Germany), methanol (Merck, Germany), hexane (Sigma-Aldrich, Germany), isopropanol (Merck, Germany), acetone (Merck, Germany), ethylacetate (Sigma-Aldrich, Germany).

### Growing conditions and lighting system

Microgreens, basil (*Ocimum basilicum* L., ‘Sweet Genovese’) and parsley (*Petroselinum crispum*) were grown in peat substrate (Profi 1, Durpeta, Lithuania) (pH 5.8–6.5) in 0.5 l plastic vessels (18x11x6 cm) for 19 days from sowing to harvest. The following amounts of nutrients were available in the substrate: N 110, P_2_O_5_ 50, K_2_O 160 (used as mg l^–1^); microelements–Fe, Mn, Cu, B, Mo, Zn (used as mg l^–1^). Depending on size and weight, 1 g of basil and 2 g of parsley seeds were seeded per vessel. Experiments were performed in (I) controlled-environment growth chambers. Day/night temperatures of 21 / 17 ± 2°C were established with a 16 h photoperiod and a relative air humidity of 50–60%. Light emitting diode (LED) lighting units were originally designed by Tamulaitis et al. [19], consisting of commercially available LED’s with emission wavelengths of blue (LXHL–LR3C, l = 447 nm), red (LXHL-LD3C, l = 638 nm and LXM3-PD01-0300, l = 665 nm; Philips Lumileds, USA) and far-red (L735- 05-AU, l = 731 nm; Epitex, Japan). The surface area under the lighting unit was approximately 0.5 m^2^. Irradiance levels, expressed as photosynthetic photon flux density (PPFD), were set in each lighting unit from 231 μmol m^-2^ s^-1^ during growth, and up to 300 μmol m^-2^ s^-1^ during 3-day treatment after altering *R_638_ or *R_665_ PPFD level, Table 1). PPFD was measured and regulated at the vessel level using a photometer–radiometer (RF-100, Sonopan, Poland).

**Table 1:** The composition of spectral components in controlled-environment growth chambers.

Treatment	Photon flux density, μmol m^-2^ s^-1^				
	447 nm	638 nm	665 nm	731 nm	Total
	B	R	R	FR	
During growth					
B,R_638_,R_665_,FR	17.6	84.4	127.4	1.6	231
3-day treatment					
B,R_638_,R_665_,FR (control)	19.0	118.4	160.4	2.2	300
B,*R_638_,R_665_,FR^1^	15.8	171.3	111.4	1.5	300
B,R_638_,*R_665_,FR^2^	14.3	79.1	204.9	1.7	300
R_638_	0.0	300	0.0	0.0	300
R_665_	0.0	0.0	300	0.0	300

^1^Increased PPFD level during 3-day treatment, further in the text B,*R_638_,R_665_,FR will be marked as *R_638_; where an * means an increased level of red (638 nm) component;

^2^Increased PPFD level during 3-day treatment, further in the text B,R_638_,*R_665_,FR will be marked as *R_665_; where an * means an increased level of red (665 nm) component;

B–blue light; R–red light; FR–far-red light.

(II) Microgreens were grown to harvest (10 days) within a greenhouse during November in Lithuania, lat. 55° N) under daylight with supplementary lighting provided by standard high-pressure sodium lamps (HPS; Philips SON-T Agro, 400 W; 16-h photoperiod). The generated PPFD of HPS lamps was approximately 90 μmol m^-2^s^-1^. The weekly-average solar radiation inside the greenhouse during the experimental period ranged from 20 to 80 μmol m^-2^s^-1^. Tree days before the pre-harvest stage (cotyledons and two true leaves), the HPS lamps were supplemented by a solid–state illuminator (16-h). The solid-state illuminator contained red AlGaInP LEDs (LUXEON^®^ III Star, model LXHL-LD3C, Philips Lumileds Lighting Company, San Jose, Cal.) with the peak wavelength of 638 nm. These were mounted on an oblong heat sink and powered using a custom-made power supply [20]. The surface area per light treatment was approximately 2.5 m^2^. The daily integrated PPFD levels produced the artificial HPS (control—300 μmol m^-2^s^-1^); and artificial HPS + 638 (treatment); HPS lamps generated 90 μmol m^-2^s^-1^ and red LEDs generated 210 μmol m^-2^s^-1^.

### Determination of total phenolic compounds

Using a calorimetric method, the total content of phenolic compounds was determined using methanol extracts of basil or parsley (1 g of plant tissues grounded with liquid nitrogen and diluted with 10 ml of 80% methanol) [21]. The extract was shaken for 30 min. and subsequently centrifuged at 2012 x g for 20 min. 1 ml of extract was diluted with 1 ml Folin-Ciocalteau reagent (Folin reagent diluted with bi-distilled water 1:10) and with 2 ml 7.5% Na_2_CO_3_ solution. The absorbance was measured after 20 min at 765 nm using a Genesys 6 spectrophotometer (Thermospectronic, USA) against distilled water as a blank. Gallic acid was used as a standard; the total phenolics were evaluated using a calibration curve.

### DPPH• radical-scavenging activity

The antioxidant activity of methanol extracts of the investigated plants was evaluated spectrophotometrically relating to the 2,2–diphenyl–1–picrylhydrazyl (DPPH^•^) free radical scavenging capacity [21]. The absorbance scanned after 16 minutes from the beginning of the reaction at 515 nm was used for the calculation of the ability of seed material to scavenge DPPH^•^ free radicals (μmol g^-1^).

### Determination of ascorbic acid

Ascorbic acid content was evaluated using a spectrophotometric method [22]. Plant tissues (1 g) was homogenized in 10 ml of 5% oxalic acid in order to avoid the loss of ascorbic acid, and subsequently centrifuged (5 min, 1691 x g). Additionally, 1 ml of extract was mixed with 2 ml of 0.1% methyl viologen and 2 ml 2 mol l^-1^ sodium hydroxide. The solution was gently shaken and allowed to stand for 2 minutes. The coloured radical ion was measured at 600 nm against the radical blank.

### Determination of tocopherols

Alpha tocopherol (α-T) content was evaluated according to Fernandez-Orozco et al. [23] using high-performance liquid chromatography (HPLC) on a Pinacle II silica column, 5 μm particle size, 150 x 4.6 mm (Restek, USA). Tocopherol homologues were extracted using pure hexane (1g of sample / 10 ml of solvent), centrifuged (5 min, 349 x g) and filtrated through 0.45 μm PTFE membrane using s syringe filter (VWR International, USA). The HPLC 10A system, equipped with RF-10A fluorescence detector (Shimadzu, Japan) was used for analysis. Peak was detected using an excitation wavelength of 295 nm and emission wavelength at 330 nm. The mobile phase was 0.5% isopropanol in hexane, flow rate 1 ml min^-1^.

### Determination of carotenoids

Contents of lutein and β-carotene were evaluated using HPLC with a diode array detector (at 440 nm), on a YMC Carotenoid column (3 μm particle size, 150 x 4.0 mm; YMC, Japan). Carotenoids were extracted using 80% acetone (1 g of sample grounded with liquid nitrogen 10 ml^-1^ of solvent), centrifuged (5 min, 349 x g), and filtrated through a 0.45-μm nylon membrane syringe filter (VWR International, USA). The HPLC 10A system (Shimadzu, Japan) equipped with a diode array (SPD-M 10A VP) detector was used for analysis. Peaks were detected at 440 nm. The mobile phase consisted of A (80% methanol, 20% water) and B (100% ethylacetate). Gradient: 0 min; 20% B, 2.5 min; 22.5% B, 20–22.5 min; 50% B, 24–26 min; 80% B, 31–34 min; 100% B, 42–47 min; and 20% B, flow rate 1 ml min^-1^. The sensitivity of all chromatographic methods was established using a method validation procedure outlined by Edelenbos et al. [24].

### Statistical analysis

Each light treatment contained four replicate vessels per species. Three biological replications of randomly selected plants (0.5–1 g per sample) were used for each analysis. Three to five analytical replications of treated antioxidants were performed for each treatment. Data analysis was processed using one-way analysis of variance (ANOVA), the Fisher’s LSD test to trial mean at the confidence level p = 0.05. Correlation coefficient (r) was evaluated using STATISTICA 7.Data was processed using MS Excel software (version 7.0).

## Results and Discussion

DPPH^•^ radical-scavenging activity and lutein accumulation showed the same reaction to lighting conditions in both basil and parsley grown in controlled-environment growth chambers (Table 2). The increased PPFD level of *R_638_ and *R_665_ LEDs increased DPPH• radical-scavenging activity by approximatelyabout 21% in basil and 13-–14% higher DPPH• radical-scavenging activity in basil and parsley respectively. In agreement with Palaniswamy and Palaniswamy [25], correlation analysis between tested antioxidants showed strong (0.54–0.73) and very strong (0.76–1.00) relationships in basil and parsley extracts (Table 3). However, statistically significant correlations (P≤0.05) were found only between DPPH^•^ and β-carotene in basil and parsley extracts under increased *R_638_; and also between DPPH^•^ and ascorbic acid under increased *R_665_ in parsley extracts. DPPH^•^ radical-scavenging activity increased by up to 15.3% under sole R_638_, and up to 21.3% under sole R_665_ LED in basil extracts. Whereas sole R_638_ LED had no significant effect on DPPH^•^ radical-scavenging activity in parsley, under sole R_665_ LED, an increase of 16.8% was detected (Table 2). Statistically significant correlations (P≤0.05) were found between DPPH^•^ and total phenols, and between lutein and β-carotene under sole R_665_ in parsley extracts (Table 3). These results correlate with a study conducted by Wu et al. [26] where seedlings cultured with red LEDs appeared to be potent in antioxidant capacity. Whereas according to Lee et al. [27], DPPH^•^ radical-scavenging activity of barley extracts was lowest under red LEDs. Our results suggest that in the control lighting treatment (B,R_638_,R_665_,FR), where *PPFD* level was increased equally all components, showed statistically significant correlations (P≤0.05) between DPPH^•^ and ascorbic acid in basil extracts, between DPPH^•^ and β-carotene, and between lutein and β-carotene in parsley (Table 3).

**Table 2:** The effect of red LED light on antioxidant contents in basil and parsley grown in (I) growth chambers and (II) greenhouse.

Treatment	DPPH,	Total phenols,	α-T,	Lutein,	β-carotene,	AA,
	μmol g^-1^	mg g^-1^	μg g^-1^	μg g^-1^	μg g^-1^	mg g^-1^
(I)	Basil					
B,R_638_,R_665_,FR	7.75	0.54	43.33	74.40	59.69	3.83
B,*R_638_,R_665_,FR^1^	9.80^A^	0.64^A^	85.00^A^	61.42^B^	33.65^B^	5.56^A^
B,R_638_,*R_665_,FR^2^	9.77^A^	0.63^A^	41.71	67.88^B^	60.11	5.85^A^
R_638_	9.15^A^	0.61^A^	48.03^A^	69.35	44.13^B^	3.63
R_665_	9.85^A^	0.63^A^	40.13	47.80^B^	40.61^B^	2.51^B^
LSD_05_	0.39	0.03	*4*.*63*	*5*.*90*	*4*.*10*	*0*.*22*
(II)						
HPS	7.96	0.95	136.03	20.57	13.97	1.06
HPS+638	10.34	1.50^A^	167.05^A^	40.17^A^	26.81^A^	1.25
LSD_05_	*6*.*60*	*0*.*36*	*9*.*27*	*2*.*21*	*1*.*86*	*0*.*9*
(I)	Parsley					
B,R_638_,R_665_,FR	5.68	0.57	577.4	106.62	46.68	13.39
B,*R_638_,R_665_,FR^1^	6.52^A^	0.50^B^	346.2^B^	76.31^B^	52.98^A^	2.61^B^
B,R_638_,*R_665_,FR^2^	6.63^A^	0.52^B^	516.4	82.68^B^	43.54	0.94^B^
R_638_	5.99	0.46^B^	581.0	90.07^B^	53.45^A^	2.73^B^
R_665_	6.83^A^	0.56	852.9^A^	99.41^B^	52.29^A^	8.41^B^
LSD_05_	*0*.*70*	*0*.*01*	*69*.*96*	*5*.*35*	*5*.*26*	*1*. *01*
(II)						
HPS	1.88	0.62	245.64	43.09	21.29	0.41
HPS+638	2.14	1.06^A^	308.38	40.48	26.69	0.35
LSD_05_	*0*.*42*	*0*.*34*	*81*.*16*	*2*.*62*	*10*.*76*	*1*.*45*

^1^Increased PPFD level during 3-day treatment, further in the text B,*R_638_,R_665_,FR will be marked as *R_638_;

^2^Increased PPFD level during 3-day treatment, further in the text B,R_638_,*R_665_,FR will be marked as *R_665_

Mean differences with the same letters are significantly (P ≤ 0.05) different from control (B,R_638_,R_665_,FR–for (I) growth chambers, and HPS–for (II) greenhouse) using Fisher’s LSD test. AA–ascorbic acid; T–tocopherol.

(I) experiment was performed in growth chambers under controlled temperature, photoperiod and spectral composition conditions.

(II) experiment was performed in greenhouse under controlled temperature, photoperiod and artificial lighting conditions. The weekly-average solar radiation inside the greenhouse during the period of the experimental period in November ranged from 20 to 80 μmol m^-2^s^-1^.

**Table 3:** Correlation analysis (r) between antioxidants (*P≤0.05).

Growth environment		(I) growth chambers					(II) greenhouse	
Treatment		C	*R_638_;	*R_665_	R_638_	R_665_	HPS	HPS+638
Correlation between		Basil						
DPPH	Total phenols	0.83	0.95	0.99	0.60	0.99	0.99	0.61
	α-T	0.97	0.81	0.99	0.98	0.65	0.99	0.96
	Lutein	0.98	0.90	0.85	0.68	0.97	1.00*	0.61
	β-carotene	0.99	1.00*	0.80	0.92	0.81	0.97	0.79
	AA	1.00*	0.80	0.81	0.99	0.95	0.89	0.99
Total phenols	α-T	0.94	0.59	1.00*	0.73	0.73	1.00*	0.80
	Lutein	0.69	0.71	0.77	0.99	0.99	0.98	1.00*
	β-carotene	0.91	0.95	0.71	0.86	0.87	1.00*	0.97
	AA	0.85	0.57	0.72	0.69	0.98	0.95	0.71
α-T	Lutein	0.89	0.99	0.78	0.80	0.82	0.98	0.80
	β-carotene	1.00	0.81	0.72	0.98	0.97	0.99	0.93
	AA	0.98	1.00*	0.73	1.00*	0.86	0.94	0.99
Lutein	β-carotene	0.93	0.89	1.00	0.91	0.93	0.96	0.97
	AA	0.97	0.98	1.00	0.77	1.00	0.86	0.71
β-carotene	AA	0.99	0.80	1.00*	0.96	0.96	0.97	0.86
		Parsley						
DPPH	Total phenols	0.96	0.98	0.95	0.78	1.00*	1.00*	1.00*
	α-T	0.83	0.94	0.86	0.71	0.92	1.00*	0.95
	Lutein	1.00	0.85	0.96	0.57	0.99	0.93	0.82
	β-carotene	1.00*	1.00*	0.92	0.95	0.98	1.00*	1.00*
	AA	0.63	0.90	1.00*	0.99	0.98	0.99	0.99*
Total phenols	α-T	0.95	0.99	0.97	0.99	0.93	1.00*	0.93
	Lutein	0.98	0.94	0.82	0.96	0.99	0.94	0.85
	β-carotene	0.97	0.97	0.99	0.94	0.99	1.00*	0.99
	AA	0.83	0.80	0.96	0.86	0.98	0.99	1.00*
α-T	Lutein	0.87	0.98	0.67	0.98	0.97	0.95	0.60
	β-carotene	0.86	0.92	0.99	0.89	0.98	1.00*	0.96
	AA	0.96	0.69	0.87	0.80	0.83	0.98	0.90
Lutein	β-carotene	1.00*	0.82	0.76	0.79	1.00*	0.92	0.79
	AA	0.70	0.54	0.95	0.68	0.94	0.87	0.88
β-carotene	AA	0.68	0.92	0.93	0.99	0.93	0.99	0.98

B,R_638_,R_665_,FR (control)–C;

Increased PPFD level during 3-day treatment, further in the text and in the table B,*R_638_,R_665_,FR will be marked as *R_638_;

Increased PPFD level during 3-day treatment, further in the text and in the table B,R_638_,*R_665_,FR will be marked as *R_665_;

(I) experiment was performed in growth chambers under controlled temperature, photoperiod and spectral composition conditions.

(II) experiment was performed in greenhouse under controlled temperature, photoperiod and artificial lighting conditions. The weekly-average solar radiation inside the greenhouse during the period of experimental period in November ranged from 20 to 80 μmol m^-2^s^-1^.

According to our data, in comparison to the controls, significantly lower amounts of lutein were observed in basil extracts under increased *R_638_ (-17.4%), increased *R_665_ (-8.8%) and sole R_665_ (-35.8%). Additionally, significantly lower amounts of lutein were observed in parsley under increased *R_638_ (-28.4%), increased *R_665_ (-22.5%), sole R_638_ (-15.5%) and sole R_665_ (-6.8%). Significantly lower amounts of β-carotene were detected in basil under increased *R_638_ (-43.6%), sole R_638_ (-23.1%) and sole R_665_ (-34.5%). Thus, reduced PPFD level of blue LEDs (about 1–1.2% from control) and increased PPFD level of *R_638_ or *R_665_ nm light resulted in significantly lower amounts of carotenoid pigments. The opposite effect of the same LED treatment was demonstrated in parsley, as significant increases of β-carotene were observed under increased *R_638_ (11.9%), sole R_638_ (12.7%) and sole R_665_ (10.7%) (Table 2). Lefsrud et al. [28] demonstrated that lutein accumulation was enhanced under red LEDs combined with fluorescent lamps; and β-carotene was enhanced–under blue LEDs combined with fluorescent lamps. Li and Kubota [6] demonstrated that carotenoid concentration decreased under farred LEDs supplemented with cool-white fluorescent lamps, but increased under blue LEDs supplemented with fluorescent lamps. According to Lefsrud et al. [29] irradiance can be a major factor in carotenoid pigment accumulation. Authors demonstrated that maximum levels of lutein, β-carotene and chlorophylls accumulation occurred at maximum absorption and decreased linearly in relation to irradiance. Conversely, low irradiance levels at 400 and 524 nm wavelengths did not significantly affect pigment accumulation and it was no different from 440 nm, although the irradiance was significantly different [28].

The response to the LED lighting conditions of other tested antioxidants differed between basil and parsley extracts. In basil extracts, the total phenolic content of significantly increased under all treatments (11.5–15.6%) in comparison to the control, whereas in parsley, total phenolic content significantly decreased under increased *R_638_ (-12.3%), increased *R_665_ (-8.8%) and sole R_638_ (-19.3%) (Table 2). Conversely, in basil extracts subjected to increased *R665 there were significant correlations between total phenols and α-tocopherol, as well as between β-carotene and ascorbic acid (Table 3). Li and Kubota [6] demonstrated that fluorescent lamps supplemented red LEDs caused an increase of phenolics in baby leaf lettuce. Kim et al. [30] found that blue LED treatment increased the amount of phenolic compounds in tomato. These data are in agreement with Johkan et al. [31] where supplemental blue light resulted in an increase of phenolic compounds in red leaf lettuce. With this in mind, the differences displayed in this study occurred due to different quality and composition of phenolic compounds in basil and parsley and they are therefore significantly influenced by the quality of light.

In basil extracts, significantly higher amounts of α-tocopherol were measured under increased *R_638_ (49.0%) and sole R_638_ (9.8%) (Table 2) and significant correlations were observed between α-tocopherol and ascorbic acid under increased *R_638_ and under sole R_638_ (Table 3). In parsley extracts,increased *R_638_ led to significant decrease of α-tocopherol content (-40.0%), sole R_665_ resulted in significant increase (32.3 (Table 2). Park et al. [32] demonstrated that under sole blue LED treatments significantly higher amounts of α-tocopherol were identified in comparison with sole blue (465 nm) or fluorescent lamps. In basil, both increased *R_638_ and *R_665_ led to significant increases of ascorbic acid (68.9% and 34.5% respectively), however there were significant decreases under sole R_665_ (-34.5%), whereas in parsley, ascorbic acid decreased due to increased *R_638_ (-80.5%), increased *R_665_ (93.0%), sole R_638_ (79.6%) and sole R_665_ (37.2%). Li et al. [17] found that concentration of ascorbic acid was the greatest under blue LEDs, therefore antioxidant protection is extended from the hydrophilic into the lipophilic thylakoid space; in this antioxidant system, tocopherol may be oxidized to the radical which is then reduced by ascorbate [33]. Furthermore, phytochemicals which are located in the thylakoid (such as tocopherol) may not only supplement in stress situations but also suggest a protective role for photosynthesis.

HPS lams supplemented by R_638_ LEDs significantly induced accumulation of total phenols (36.7%), α-tocopherols (18.6%), lutein (48.8%) and β-carotene (47.9%) in basil extracts, while only significant increase of total phenolic content (41.5%) in parsley extract were detected (Table 2). These data are in agreement with results published by other authors, in which the antioxidant contents were shown to be greatly increased in various plants treated with supplemental red LED light [6, 15, 16, 28]. Moreover, in basil extracts, statistically significant correlations (P≤0.05) were found between DPPH^•^ and lutein, total phenols and α-tocopherol, total phenols and β-carotene; and in parsley extracts under HPS lighting, statistically significant correlations (P≤0.05) between DPPH^•^ and total phenols, DPPH^•^ and α-tocopherol, DPPH^•^ and β- carotene, total phenols and α-tocopherol, total phenols and β-carotene, α-tocopherol and β-carotene. Supplemental R_638_ LED lighting influenced significant correlations (P≤0.05) between total phenols and lutein in basil extracts; and between DPPH^•^ and total phenols, DPPH^•^ and β- carotene, DPPH^•^ and ascorbic acid, total phenols and ascorbic acid in parsley extracts (Table 2). Strong or very strong correlations between DPPH^•^ free-radical scavenging capacities and phenolic compounds indicates that the presence of the phenolic compounds depends upon the antioxidant capacity [34]. High correlations between antioxidants support the statement that lower concentrations of some antioxidants in the plant, causes an increase of other antioxidants [35].

## Conclusions

In general, under supplemental or increased red 638 nm light amounts of tested antioxidants were greater in basil, whereas in parsley sole 665 nm or sole 638 nm light is more favourable. Increased or supplemental red light significantly increased contents of phenolics, α-tocopherol, ascorbic acid and DPPH^•^ but suppressed the accumulation of lutein and β-carotene in basil; whereas an increase of and β-carotene and DPPH^•^ was observed in parsley. It is therefore concluded, that due to the photoresponse of antioxidant compounds observed here, the photoprotective mechanism is stimulated by both light-dose and wavelength dependent reactions.

## Supporting Information

S1 Table The composition of spectral components in controlled-environment growth chambers. (DOCX)

S2 Table The effect of red LED light on antioxidant contents in basil and parsley grown in (I) growth chambers and (II) greenhouse. (DOCX)

S3 Table Correlation analysis (r) between antioxidants (*P≤0.05). (DOCX)
