# Supplementary material for: Red Light-Dose or Wavelength-Dependent Photoresponse of Antioxidants in Herb Microgreens
Source: PLoS One. 2016 Sep 27;11(9):e0163405. doi: 10.1371/journal.pone.0163405 (PMC5038936; doi:10.1371/journal.pone.0163405)
Supplement: S1 Table — (DOCX) [file pone.0163405.s001.docx]

**Table 1 in S1** The composition of spectral components in controlled-environment growth chambers.

| Treatment | Photon flux density, μmol m^-2^ s^-1^ | | | | | |
| --- | --- | --- | --- | --- | --- | --- |
|  | 447 nm | | 638 nm | 665 nm | 731 nm | Total |
|  | B | | R | R | FR |  |
| During growth | | | | | | |
| B,R_638_,R_665_,FR | 17.6 | | 84.4 | 127.4 | 1.6 | 231 |
| 3-day treatment | | | | | | |
| B,R_638_,R_665_,FR (control) | | 19.0 | 118.4 | 160.4 | 2.2 | 300 |
| B,*R_638_,R_665_,FR^1^ | | 15.8 | 171.3* | 111.4 | 1.5 | 300 |
| B,R_638_,*R_665_,FR^2^ | | 14.3 | 79.1 | 204.9* | 1.7 | 300 |
| R_638_ | | 0.0 | 300 | 0.0 | 0.0 | 300 |
| R_665_ | | 0.0 | 0.0 | 300 | 0.0 | 300 |

^1^Increased *PPFD* level during 3-day treatment, further in the text B,*R_638_,R_665_,FR will be marked as *R_638_;

^2^Increased *PPFD* level during 3-day treatment, further in the text B,R_638_,*R_665_,FR will be marked as *R_665_

B – blue light; R – red light; FR – far-red light.
