# Supplementary material for: Red Light-Dose or Wavelength-Dependent Photoresponse of Antioxidants in Herb Microgreens
Source: PLoS One. 2016 Sep 27;11(9):e0163405. doi: 10.1371/journal.pone.0163405 (PMC5038936; doi:10.1371/journal.pone.0163405)
Supplement: S2 Table — (DOCX) [file pone.0163405.s002.docx]

**Table 2 in** **S2** The effect of red LED light on antioxidant contents in basil and parsley grown in (I) growth chambers and (II) greenhouse

| Treatment | DPPH, | | | Total phenols, | α-T, | Lutein, | β-carotene, | AA, | |
| --- | --- | --- | --- | --- | --- | --- | --- | --- | --- |
|  | µmol g^-1^ | | | mg g^-1^ | µg g^-1^ | µg g^-1^ | µg g^-1^ | mg g^-1^ | |
| (I) | | | Basil | | | | | |  |
| B,R_638_,R_665_,FR | | 7.75 | | 0.54 | 43.33 | 74.40 | 59.69 | 3.83 | |
| B,*R_638_,R_665_,FR^1^ | | 9.80^A^ | | 0.64^A^ | 85.00^A^ | 61.42^B^ | 33.65^B^ | 5.56^A^ | |
| B,R_638_,*R_665_,FR^2^ | | 9.77^A^ | | 0.63^A^ | 41.71 | 67.88^B^ | 60.11 | 5.85^A^ | |
| R_638_ | | 9.15^A^ | | 0.61^A^ | 48.03^A^ | 69.35 | 44.13^B^ | 3.63 | |
| R_665_ | | 9.85^A^ | | 0.63^A^ | 40.13 | 47.80^B^ | 40.61^B^ | 2.51^B^ | |
| LSD_05_ | | 0.39 | | 0.03 | *4.63* | *5.90* | *4.10* | 0.22 | |
| (II) | |  | |  |  |  |  |  | |
| HPS | | 7.96 | | 0.95 | 136.03 | 20.57 | 13.97 | 1.06 | |
| HPS+638 | | 10.34 | | 1.50^A^ | 167.05^A^ | 40.17^A^ | 26.81^A^ | 1.25 | |
| LSD_05_ | | *6.60* | | *0.36* | *9.27* | *2.21* | *1.86* | *0.9* | |
| (I) | | | Parsley | | | | | |  |
| B,R_638_,R_665_,FR | | 5.68 | | 0.57 | 577.4 | 106.62 | 46.68 | 13.39 | |
| B,*R_638_,R_665_,FR^1^ | | 6.52^A^ | | 0.50^B^ | 346.2^B^ | 76.31^B^ | 52.98^A^ | 2.61^B^ | |
| B,R_638_,*R_665_,FR^2^ | | 6.63^A^ | | 0.52^B^ | 516.4 | 82.68^B^ | 43.54 | 0.94^B^ | |
| R_638_ | | 5.99 | | 0.46^B^ | 581.0 | 90.07^B^ | 53.45^A^ | 2.73^B^ | |
| R_665_ | | 6.83^A^ | | 0.56 | 852.9^A^ | 99.41^B^ | 52.29^A^ | 8.41^B^ | |
| LSD_05_ | | *0.70* | | *0.01* | *69.96* | *5.35* | *5.26* | *1. 01* | |
| (II) | |  | |  |  |  |  |  | |
| HPS | | 1.88 | | 0.62 | 245.64 | 43.09 | 21.29 | 0.41 | |
| HPS+638 | | 2.14 | | 1.06^A^ | 308.38 | 40.48 | 26.69 | 0.35 | |
| LSD_05_ | | *0.42* | | *0.34* | *81.16* | *2.62* | *10.76* | *1.45* | |

^1^Increased *PPFD* level during 3-day treatment, further in the text B,*R_638_,R_665_,FR will be marked as *R_638_;

^2^Increased *PPFD* level during 3-day treatment, further in the text B,R_638_,*R_665_,FR will be marked as *R_665_

I) experiment was performed in growth chambers under controlled temperature, photoperiod and spectral composition conditions.

(II) experiment was performed in greenhouse under controlled temperature, photoperiod and artificial lighting conditions. The weekly-average solar radiation inside the greenhouse during the period of the experimental period in November ranged from 20 to 80 µmol m^-2^s^-1^ .
