# Supplementary material for: Red Light-Dose or Wavelength-Dependent Photoresponse of Antioxidants in Herb Microgreens
Source: PLoS One. 2016 Sep 27;11(9):e0163405. doi: 10.1371/journal.pone.0163405 (PMC5038936; doi:10.1371/journal.pone.0163405)
Supplement: S3 Table — (DOCX) [file pone.0163405.s003.docx]

**Table 3 in S3** Correlation analysis (r) between antioxidants (*P≤0.05)

| Growth environment | | (I) growth chambers | | | | | | (II) greenhouse | |
| --- | --- | --- | --- | --- | --- | --- | --- | --- | --- |
| Treatment | | C | *R_638_ | *R_665_ | | R_638_ | R_665_ | HPS | HPS+638 |
| Correlation between | | Basil | | | | | | | |
| DPPH | Total phenols | 0.83 | 0.95 | 0.99 | 0.60 | | 0.99 | 0.99 | 0.61 |
|  | α-T | 0.97 | 0.81 | 0.99 | 0.98 | | 0.65 | 0.99 | 0.96 |
|  | Lutein | 0.98 | 0.90 | 0.85 | 0.68 | | 0.97 | 1.00* | 0.61 |
|  | β-carotene | 0.99 | 1.00* | 0.80 | 0.92 | | 0.81 | 0.97 | 0.79 |
|  | AA | 1.00* | 0.80 | 0.81 | 0.99 | | 0.95 | 0.89 | 0.99 |
| Total phenols | α-T | 0.94 | 0.59 | 1.00* | 0.73 | | 0.73 | 1.00* | 0.80 |
|  | Lutein | 0.69 | 0.71 | 0.77 | 0.99 | | 0.99 | 0.98 | 1.00* |
|  | β-carotene | 0.91 | 0.95 | 0.71 | 0.86 | | 0.87 | 1.00* | 0.97 |
|  | AA | 0.85 | 0.57 | 0.72 | 0.69 | | 0.98 | 0.95 | 0.71 |
| α-T | Lutein | 0.89 | 0.99 | 0.78 | 0.80 | | 0.82 | 0.98 | 0.80 |
|  | β-carotene | 1.00 | 0.81 | 0.72 | 0.98 | | 0.97 | 0.99 | 0.93 |
|  | AA | 0.98 | 1.00* | 0.73 | 1.00* | | 0.86 | 0.94 | 0.99 |
| Lutein | β-carotene | 0.93 | 0.89 | 1.00 | 0.91 | | 0.93 | 0.96 | 0.97 |
|  | AA | 0.97 | 0.98 | 1.00 | 0.77 | | 1.00 | 0.86 | 0.71 |
| β-carotene | AA | 0.99 | 0.80 | 1.00* | 0.96 | | 0.96 | 0.97 | 0.86 |
|  |  | Parsley | | | | | | | |
| DPPH | Total phenols | 0.96 | 0.98 | 0.95 | 0.78 | | 1.00* | 1.00* | 1.00* |
|  | α-T | 0.83 | 0.94 | 0.86 | 0.71 | | 0.92 | 1.00* | 0.95 |
|  | Lutein | 1.00 | 0.85 | 0.96 | 0.57 | | 0.99 | 0.93 | 0.82 |
|  | β-carotene | 1.00* | 1.00* | 0.92 | 0.95 | | 0.98 | 1.00* | 1.00* |
|  | AA | 0.63 | 0.90 | 1.00* | 0.99 | | 0.98 | 0.99 | 0.99* |
| Total phenols | α-T | 0.95 | 0.99 | 0.97 | 0.99 | | 0.93 | 1.00* | 0.93 |
|  | Lutein | 0.98 | 0.94 | 0.82 | 0.96 | | 0.99 | 0.94 | 0.85 |
|  | β-carotene | 0.97 | 0.97 | 0.99 | 0.94 | | 0.99 | 1.00* | 0.99 |
|  | AA | 0.83 | 0.80 | 0.96 | 0.86 | | 0.98 | 0.99 | 1.00* |
| α-T | Lutein | 0.87 | 0.98 | 0.67 | 0.98 | | 0.97 | 0.95 | 0.60 |
|  | β-carotene | 0.86 | 0.92 | 0.99 | 0.89 | | 0.98 | 1.00* | 0.96 |
|  | AA | 0.96 | 0.69 | 0.87 | 0.80 | | 0.83 | 0.98 | 0.90 |
| Lutein | β-carotene | 1.00* | 0.82 | 0.76 | 0.79 | | 1.00* | 0.92 | 0.79 |
|  | AA | 0.70 | 0.54 | 0.95 | 0.68 | | 0.94 | 0.87 | 0.88 |
| β-carotene | AA | 0.68 | 0.92 | 0.93 | 0.99 | | 0.93 | 0.99 | 0.98 |

Mean differences with the same letters are significantly (P ≤ 0.05) different from control (B,R_638_,R_665_,FR – for (I) growth chambers, and HPS – for (II) greenhouse) using Fisher’s LSD test. AA – ascorbic acid; T – tocopherol.

B,R_638_,R_665_,FR (control) – C;

B,*R_638_,R_665_,FR^1^ - *R_638_;

B,R_638_,*R_665_,FR^2^ - *R_665_;

(I) experiment was performed in growth chambers under controlled temperature, photoperiod and spectral composition conditions.

(II) experiment was performed in greenhouse under controlled temperature, photoperiod and artificial lighting conditions. The weekly-average solar radiation inside the greenhouse during the period of the experimental period in November ranged from 20 to 80 µmol m^-2^s^-1^.
